# Flower Colours through the Lens: Quantitative Measurement with Visible and Ultraviolet Digital Photography

**DOI:** 10.1371/journal.pone.0096646

**Published:** 2014-05-14

**Authors:** Jair E. Garcia, Andrew D. Greentree, Mani Shrestha, Alan Dorin, Adrian G. Dyer

**Affiliations:** 1 School of Media and Communication, RMIT University, Melbourne, Victoria, Australia; 2 School of Applied Sciences, RMIT University, Melbourne, Victoria, Australia; 3 Faculty of Information Technology, Monash University, Clayton, Victoria, Australia; University of Sussex, United Kingdom

## Abstract

**Background:**

The study of the signal-receiver relationship between flowering plants and pollinators requires a capacity to accurately map both the spectral and spatial components of a signal in relation to the perceptual abilities of potential pollinators. Spectrophotometers can typically recover high resolution spectral data, but the spatial component is difficult to record simultaneously. A technique allowing for an accurate measurement of the spatial component in addition to the spectral factor of the signal is highly desirable.

**Methodology/Principal findings:**

Consumer-level digital cameras potentially provide access to both colour and spatial information, but they are constrained by their non-linear response. We present a robust methodology for recovering linear values from two different camera models: one sensitive to ultraviolet (UV) radiation and another to visible wavelengths. We test responses by imaging eight different plant species varying in shape, size and in the amount of energy reflected across the UV and visible regions of the spectrum, and compare the recovery of spectral data to spectrophotometer measurements. There is often a good agreement of spectral data, although when the pattern on a flower surface is complex a spectrophotometer may underestimate the variability of the signal as would be viewed by an animal visual system.

**Conclusion:**

Digital imaging presents a significant new opportunity to reliably map flower colours to understand the complexity of these signals as perceived by potential pollinators. Compared to spectrophotometer measurements, digital images can better represent the spatio-chromatic signal variability that would likely be perceived by the visual system of an animal, and should expand the possibilities for data collection in complex, natural conditions. However, and in spite of its advantages, the accuracy of the spectral information recovered from camera responses is subject to variations in the uncertainty levels, with larger uncertainties associated with low radiance levels.

## Introduction

The pollination of flowering plants (angiosperms) is often facilitated by animal vectors including bees and birds [1], [2]. In many cases, pollination vectors are attracted to visit flowers to collect small nutritional rewards, and thus incidentally transfer pollen between flowers of the same species to effect sexual reproduction [3], [4]. This model of plant reproduction has been well studied since the time of Darwin [5] and has received considerable attention from ecologists, botanists and evolutionary biologists [6]. Early researchers already appreciated that animals may have different visual perception to humans [7]–[10] and both early film-based photographic [11]–[13] and spectrophotometer [14] recordings revealed the presence of UV signals from some flowers [15]. Since the publication of the influential book: ‘The Ecology of Vision’ [16], there has been strong interest in collecting empirical data to understand the signal-receiver relationship between important pollinators such as bees and flowering plants [3].

The accurate quantification of the physical component of biological signals has been facilitated by improvements in spectrophotometers and spectroradiometers [17]. Furthermore, using these tools a strong fit has been found between the visual discrimination abilities of bees and the spectral reflectance characteristics of flowering plants visited by them [18]–[21], as well as between bird vision and the spectral reflectance of flowers only visited by birds [22]. However, in spite of the portability and accuracy obtainable from modern spectrophotometers, these instruments are often limited to measurements from a small number of sample points on a surface [17], [19]. This is potentially a limitation in plant-pollinator studies since many flowers contain complex colour patterns [23] and important pollinators like honeybees can simultaneously perceive both colour and shape information for making decisions about rewarding flowers [24], [25]. These previous findings therefore strongly suggest a need to accurately map the spatial (2-dimensional) aspects of flower colour patterns.

The popularisation in recent times of digital single lens reflex (DSLR) cameras equipped with three different colour filters: ‘red’, ‘green’ and ‘blue’ (RGB), has inspired the development of methodologies for quantifying flower colours directly from camera responses [23]. However as responses from most consumer-level digital cameras are not constrained to provide an accurate measurement of the amount of energy reflected by a given object, but to produce a visually appealing representation of the recorded subject [26], [27], unprocessed camera responses can not be used for quantitative image analysis. In order to use camera responses as measures of colour [28]–[30], linear camera responses must first be recovered from the original, non-linear responses returned from the camera before measuring incident irradiation from RGB values at the pixel level [26], [31].

RGB responses corresponding to signals reflecting radiation in the UV region of the spectrum are also known to show a non-linear response in two tested camera models [32], [33]. Therefore, in order to make use of the extended spectral sensitivity of specialised digital cameras into the UV region, their responses should be linearised as well. Fortunately, the same principles involved in the linearisation of responses of digital cameras sensitive to visible radiation also apply to those obtained from UV-sensitive cameras [31]; thus once linearised, it is possible to make use of digital imaging for measuring and studying plant-pollinator visual signals in a spectral range of about 320 to 700 nm, which is visible to many common pollinators [22], [34], [35].

Previous studies have made use of linear camera responses to quantitatively characterise animal colour patterns in studies of camouflage and behaviour in the visible [26], [36] and UV regions of the electromagnetic spectrum [32], [37]; however, there is a paucity of information detailing the use of this methodology in plant studies. Recently, one study proposed the use of digital photography as a tool for the characterisation of plant signals in studies of diversity, conservation and plant-pollinator relationships [23]. However, the approach it described is limited to expressing camera responses in a purely human-based colorimetric system which significantly differs from the way most insect pollinators perceive flowers [15], [34]. Specifically, Lutz [38], described the presence of UV-reflective elements in various flower species independently from their appearance in the human visible region of the spectrum. Since then, several authors have reported more plant species whose flowers present UV-reflective elements using film-based UV photography [15], [39]–[41], and thus contribute to overall bee colour perception [15].

Here we address some of the limitations of directly using images as typically recorded by a camera for quantifying flower colours. We present a robust methodology for quantifying floral visual signals containing visible and UV components that employs physically-meaningful units from RGB camera responses. We then compare recovered reflectance values against theoretical values calculated from standard spectrophotometric measurements and the spectral sensitivity curves of the employed cameras.

## Materials and Methods

### Background and Definitions

Light reflected by an object and received on a photoreceptor produces a signal response 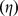, which is a function of the spectral sensitivity of the receptor *(S)*, the spectral power distribution of the illumination *(E)* and the reflectance spectrum *(R)* of the illuminated object expressed as:
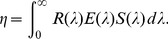
(1)
Equation 1 is the core of most colourimetric equations replacing *(S)* by a function describing the spectral sensitivity of one of the three different cone classes in the human visual system [42]. Equation 1 can also be applied to model photon catches by non-human visual systems [34], [43].

When Equation 1 is applied to model the response of a CCD or CMOS digital camera, the camera response (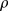) is expressed in terms of pixel intensity levels. At any given pixel, the camera response is a function of: the sensor response (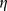), the intensity of the signal *(I)*, the selected exposure parameters *(H)*, including selected aperture, integration time, image magnification and sensor size [44], and the amplification (gain) of the sensor response *(G)*; a nontrivial function of *I* and *H*, which is unique to each camera model and colour channel [26], [31]. In the case of an RGB device this relation is expressed as:
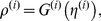
(2)for each colour channel *i*.

In digital cameras for technical use, *G* is a constant, thus the camera response is linearly related to the intensity of the signal, which in turn is controlled by the exposure settings. Hence we can write Equation 2 as: 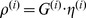. However, more typically, *G* takes a non-linear form in RGB digital cameras producing camera responses (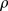) which are not lineary related to signal intensity [44]. Moreover, consumer-level camera responses are also subject to other non-linear operations, commonly referred to as ‘gamma correction’ [26]. These aim to improve the aesthetic appearance of the images when displayed on computer monitors and/or to increase the effective dynamic range of the camera to human perception [27], further contributing to the non-linear behaviour of 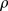 [26], [27]. Therefore, the parameter *G* in Equation 2 models most of the non-linear operations introduced into the RGB camera response [31].

When employing a digital camera to accurately quantify a visual signal by means of Equation 2, it is essential to recover the linear sensor response as a first step prior to any further analysis. This is achieved by finding a non-linear mathematical expression describing the *G* function and inverting it, implementing either analytical or optimisation methods [26], [31]. However, different camera models may have different transfer functions and the precise values of the coefficients describing the transfer function for a particular camera must be found experimentally through a characterisation and linearisation exercise. Details of different camera characterisation methodologies and fitting functions are available elsewhere (e.g. [31]), along with a precise mathematical formulation of the procedure.

### Camera System

We used a typical, consumer-level digital single lens reflex (DSLR) camera: a Canon D40 (Canon Inc., Japan) and a Fuji S3 UVIR (Fujifilm Corp, USA) DSLR modified for ultraviolet and infrared imaging to record flower reflection within a spectral interval from about 320 to 710 nm. Canon Macro Lite (Canon Inc., Japan) and Nikon Speedlight SB-14 (Nikon Corp., Japan) electronic flash units were employed as illumination due to the close resemblance of their spectral output to that of daylight [45]. The Speedlight unit was modified by an expert camera technician (Beyond Visible, USA) to increase its UV output (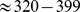 nm), thus facilitating the use of this unit in the field. To prevent radiation longer than about 395 nm from reaching the sensor of the Fuji S3 UVIR camera, a Baader U filter (Company Seven, USA) was fitted in front of a Micro Nikkor 105 quartz lens by means of a filter holder. The use of quartz optics ensured the transmission of ultraviolet radiation down to about 200 nm [33]. Flower visual signals within the visible region of the spectrum (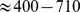 nm) were recorded with the Canon D40 camera. The camera was equipped with a Canon 100 mm Electro-Focus (EF) lens (Canon Inc., Japan) which was equipped with a skylight filter (Hoya, Philippines) for cutting-off radiation below about 390 nm.

**Figure 1:**
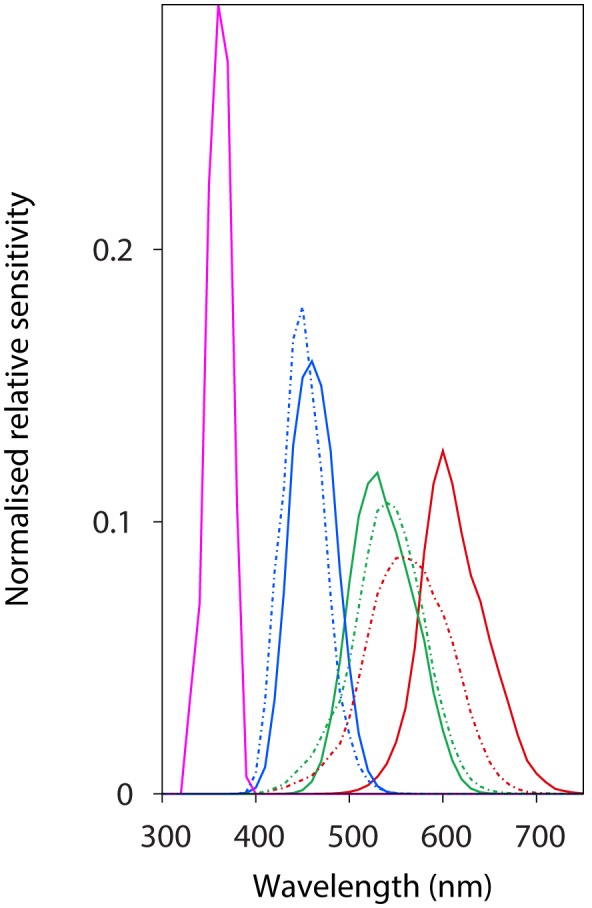
Spectral sensitivities corresponding to the red, green and blue colour channels of a Canon D40 camera (solid lines) and the UV-sensitive red channel of a Fuji S3 UVIR digital camera (magenta solid line), along with the long (red dashed line), medium (green dashed line) and short (blue dashed line) human photoreceptors [61]. Spectral sensitivities were normalised by dividing the sensitivity at each 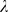 by the total area under each channel/photoreceptor curve.



Characterisation methods and reconstructed spectral sensitivity curves for the Fuji S3 UVIR camera have been published elsewhere [33]. This methodology was employed here to reconstruct the spectral sensitivity curves of the red, green and blue channels of the Canon 40D digital camera (Figure 1). Reconstructed linear spectral sensitivity functions of the two cameras were modelled fitting a Gaussian function including either one or two exponential terms:

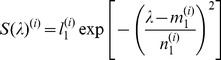



or
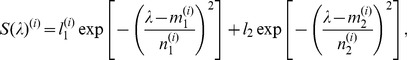
(3)for the 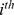 colour channel available on each camera. We chose the form of Equation 3 which best fits the sensitivity data for each colour channel based on the statistical significance of each individual parameter. Mean values and 95% confidence intervals for the obtained coefficients are provided in Table 1.

**Table 1:** Coefficients for Gaussian functions (Equation 2) fitting the spectral sensitivity curves of the ‘red’, ‘green’, and ‘blue’ channels of a Canon 40D camera and the UV-sensitive ‘red’ channel of a Fuji S3 UVIR digital camera.

	Canon 40D			Fuji S3 UVIR
	‘red’	‘green’	‘blue’	UV-‘red’
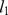	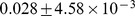	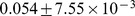	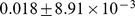	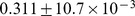
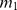	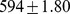	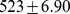	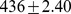	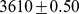
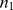	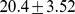	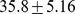	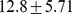	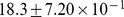
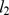	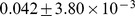	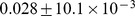	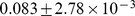	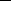
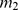	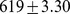	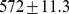	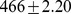	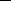
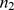	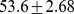	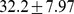	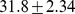	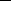

### Image Recording

We recorded images in the visible and ultraviolet region of the spectrum from flowering plants belonging to the species: *Oxalis pes-caprae, Goodenia ovata, Geranium sp., Malus domesticus, Gazania rigens, Freesia laxa, Sonchus oleraceus*, and *Eremophila macculata*, which were available at the campus of Monash University (Clayton, Victoria, Australia), during mid-spring of 2013. The use of electronic flash units with known spectral power distributions as irradiation sources reduced potential effects of variations in ambient illumination. Images were always recorded in a shadowed area, and exposure was set to minimise the contribution of ambient light to each exposure.

**Figure 2:**
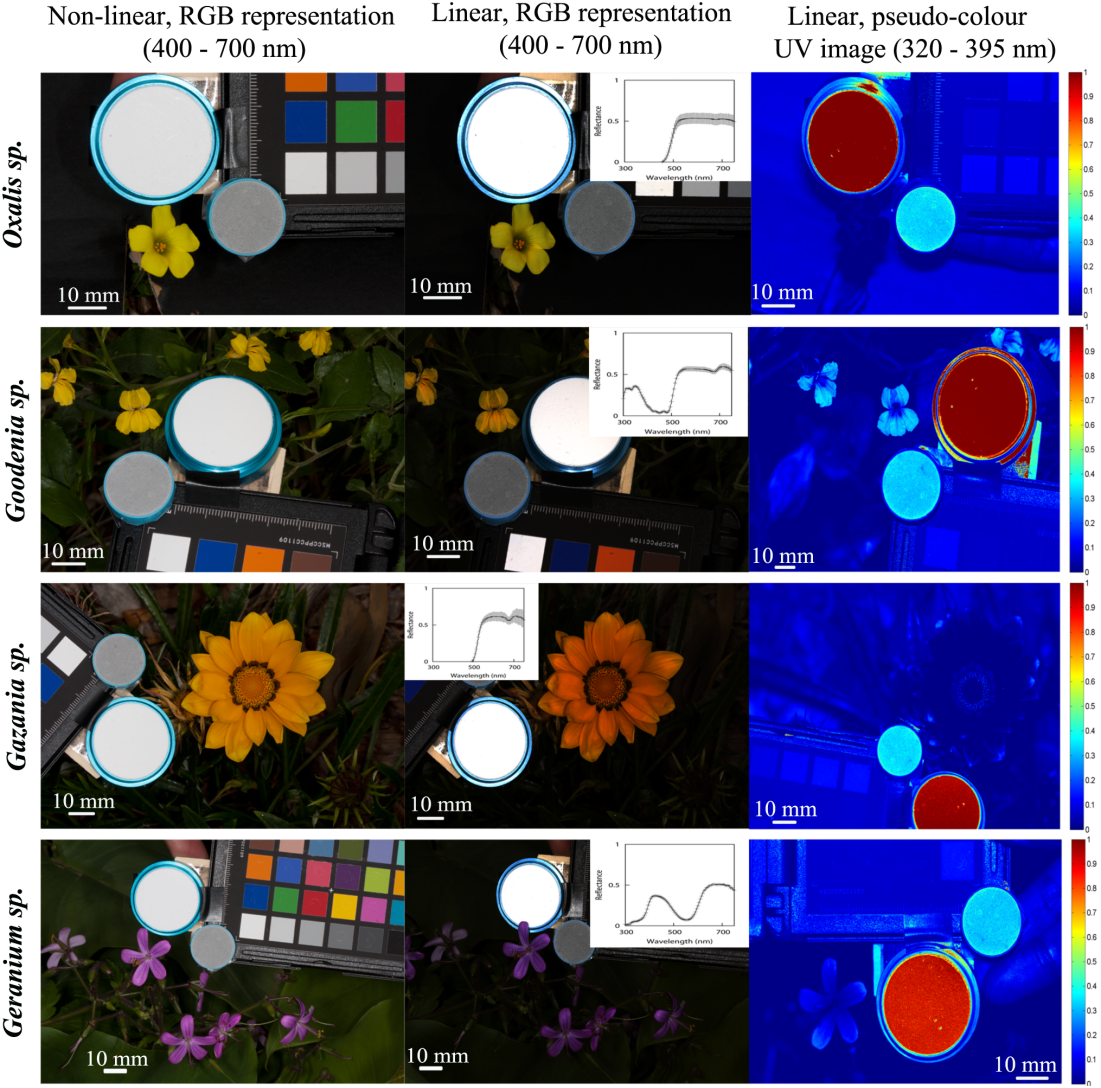
Standard, non-linear, digital images of flowers belonging to the species *Oxalis pes-caprae, Goodenia ovata, Gazania rigens* and *Geranium sp.* in the visible region of the electromagnetic spectrum (first column); reconstructed images representing the linear camera response in the visible region of the electromagnetic spectrum as recorded by the red, green and blue colour channels of a Canon 40D camera (second column); and, pseudo-colour representations of reconstructed images representing the linear camera response in the UV region of the spectrum (third column) as recorded by the ‘red’ UV-sensitive channel of a Fuji S3 UVIR camera. Second column insert depicts the mean spectral reflectance of three readings taken at the tip, middle and bottom of a single petal to account for spatio-chromatic variability within a single flower [22]. Included on each image is a white reflectance standard for spectrophotometry (large circle) and a grey achromatic standard reflecting about 33% of incident radiation. Error bars on the reflectance spectra represent one standard deviation in all cases.

**Figure 3:**
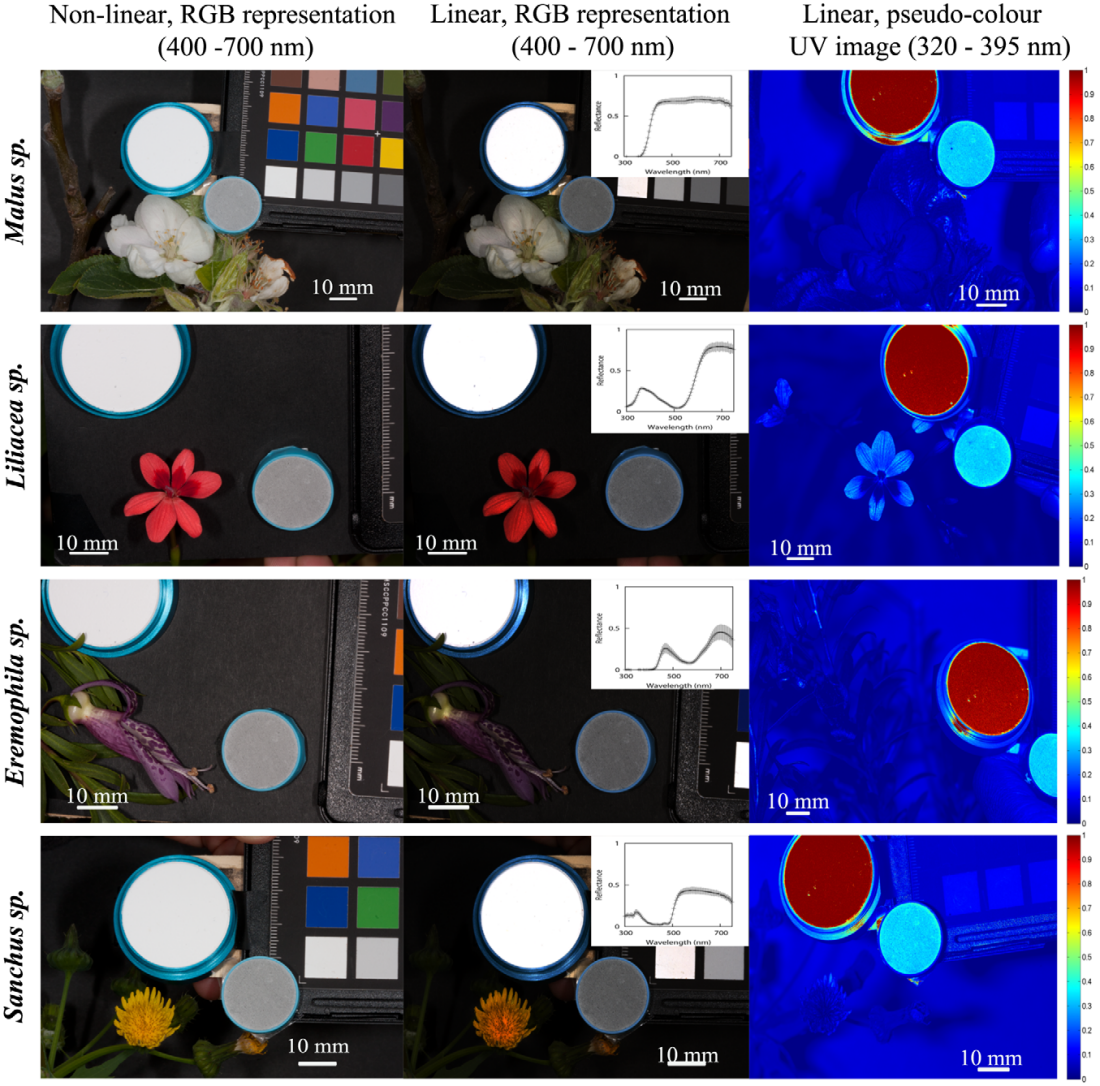
Standard, non-linear, digital images of flowers belonging to the species *Malus domestivus, Freesia laxa, Eremophila macculata* and *Sonchus oleraceus* in the visible region of the electromagnetic spectrum (first column); reconstructed images representing the linear camera response in the visible region of the electromagnetic spectrum as recorded by the red, green and blue colour channels of a Canon 40D camera (second column); and, pseudo-colour representations of reconstructed images representing the linear camera response in the UV region of the spectrum (third column) as recorded by the ‘red’ UV-sensitive channel of a Fuji S3 UVIR camera. Second column insert depicts the mean spectral reflectance of three readings taken at the tip, middle and bottom of a single petal to account for spatio-chromatic variability within a single flower [22]. Included on each image are a white reflectance standard for spectrophotometry (large circle) and a grey achromatic standard reflecting about 33% of incident radiation. Error bars as per Figure 4. *Note*: Images in row 2 column 1–2 are rotated relative to the image in row 2 column 3.

Flowers were first recorded using the ultraviolet-sensitive camera and immediately after, with the camera sensitive to visible radiation. Images were recorded with magnification ratios between 1∶3 and 1∶7 as indicated by the size scale included on each panel of Figures 2 and 3. ISO was set at 200 on both cameras. To account for image registration, the same image magnification ratio was fixed on both the Nikon and Canon lenses prior to photographing each one of the flower samples. Focus was achieved in all images by carefully positioning each one of the cameras.

**Figure 4:**
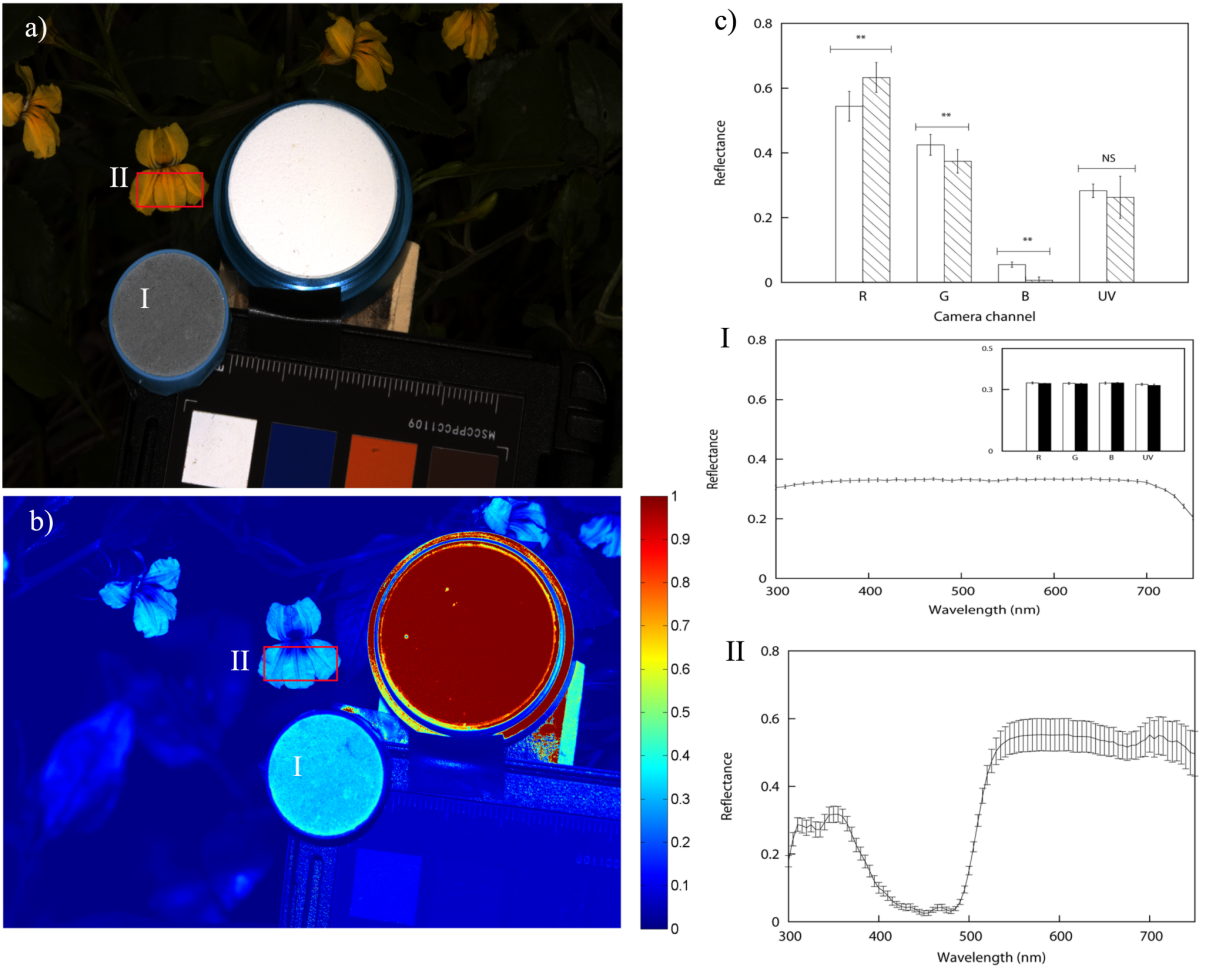
Images representing the recovered linear response of *Goodenia ovata* as recorded by a Canon 40D camera sensitive to visible radiation (panel a) and a Fuji S3 UVIR camera sensitive to UV radiation (panel b). Panel c) summarises the total reflectance recorded by the red, green, blue and ‘red’ UV-sensitive channels of the two cameras (hatched bars), and the predicted total reflectance recorded by each colour channel (white bars) along with the results of their statistical comparison. Predicted camera responses were calculated by applying Equation 1 to 15 independent spectrophotometric readings taken across the lower petals of the floral sample (graphically summarised in panel II). Panel I depicts mean reflectance spectrum, predicted and actual camera responses (Panel I insert) for an achromatic grey sample used as exposure control. Error bars represent standard deviation in all cases. 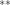
P-value significant at 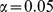; NS P-value not significant at 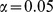. Refer to text for details.

Three different calibration targets were included on each frame as reference for setting an adequate photographic exposure: *i*) a NIST traceable white reflectance standard for spectrophotometry (Ocean Optics, USA), *ii*) a grey achromatic target made of barium sulphate and activated charcoal uniformly reflecting about 33% of incident radiation within a 300 to 400 nm spectral interval [46], (Figure 4 panel I), and *iii*) the ‘white’ target of a Passport Colour Checker (Xrite, USA) uniformly reflecting about 95% of visible radiation within a 400 to 710 nm interval [47].

Images were recorded on the native RAW format for each camera: CR2 for the Canon camera and RAF for the Fuji S3 UVIR camera. RAW image files were processed employing the Camera Raw plug-in v.6.3 (Adobe, Inc., USA) for Photoshop CS5 (Adobe, Inc,. USA). Processed RAW files were encoded into uncompressed 8-bit TIFF files using the same software package, and subsequently linearised using custom codes written for Matlab release 2012b (The Mathworks, USA) [31]. Matlab m-code is available from the authors by request.

### Image Processing, Linearisation and Segmentation

Exposure of each individual image was standardised based on the camera response predicted by Equation 1. For the exposure calculations we used reflectance spectra corresponding to the achromatic calibration targets. The CIE daylight illuminant at 6500 K (D65) was selected as reference illumination *(E)* for all calculations. Spectral power distribution for the selected illuminant was calculated following the CIE method [42].

White balance was set at 5100 K for the RGB images recorded with the Canon camera and left as interpreted by the ‘daylight’ setting available on the Fuji S3 UVIR camera. All images were encoded in the Adobe RGB 1998 colour space [48].

Processed TIFF files were linearised using look-up tables (LUT) constructed by inverting a biexponential function of the form:
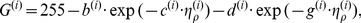
(4)which describes a readily-applicable version of the function for the different transfer functions for each one of the 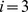 colour channels of both cameras [31]. For the particular case of the *i* = ‘red’ UV-sensitive channel of the Fuji S3 UVIR, a function with a single exponential term of the form:
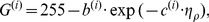
(5)with coefficients and 95% confidence bounds: 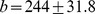 and 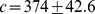 was selected to fit the transfer function. However, functions like Equation 5 do not necessarily fit transfer functions for other colour channels and/or camera models as is the case with the Canon 40D, whose transfer functions are only accurately fitted by including a second exponential term as in Equation 4 [31].

In order to recover reflectance rather than radiance values from linear RGB camera responses, the linear responses were divided by the exposure value required to obtain a maximum camera response value equal to 245 intensity levels. Selecting a maximum camera response value below the maximum 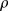 value attainable for an 8-bit colour space, *i.e.* 256 pixel intensity levels, ensured that average camera responses did not include overexposed (‘clipped’) pixel data points [26]. Total irradiance (intensity) values required to obtain 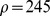 for each colour were: 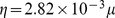
mol, 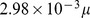
mol, 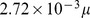
mol, for the red, green and blue channels of the Canon camera and 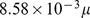
mol for the red UV-sensitive channel of the Fuji S3 UVIR camera. Exposure was calibrated individually for each channel to avoid using software-based white balancing algorithms.

### Spectrophotometric Measurements and Technique Comparison

Spectral reflectance data from the selected flower species were recorded using an Ocean Optics spectrophotometer (Ocean Optics, USA) equipped with a PX-2 pulsed xenon light source (Ocean Optics, USA). A UV-reflecting white standard from the same manufacturer was used for calibration. To account for the multiple colours displayed by the selected flowers, we measured reflectance from three different points along the major axis of each petal [19], [22]. A larger spectrophotometric sample, including 15 pseudo-randomly allocated points, was taken from a *Goodenia ovata* flower to better gauge the amount of chromatic variability. The increased sampling area enclosed the three bottom petals of the flower (Figure 4, panel b).

Camera responses (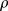) predicted by Equation 1 were compared against 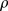 values obtained from two different image sampling schemes: *i) point sampling* and *ii) local variability* to a) estimate the magnitude of the differences expected between the photographic and spectrophotometric methods and, b) assess the amount of chromatic variability unaccounted for when point samples rather than larger areas are used to model the visual appearance of flowers.

**Figure 5:**
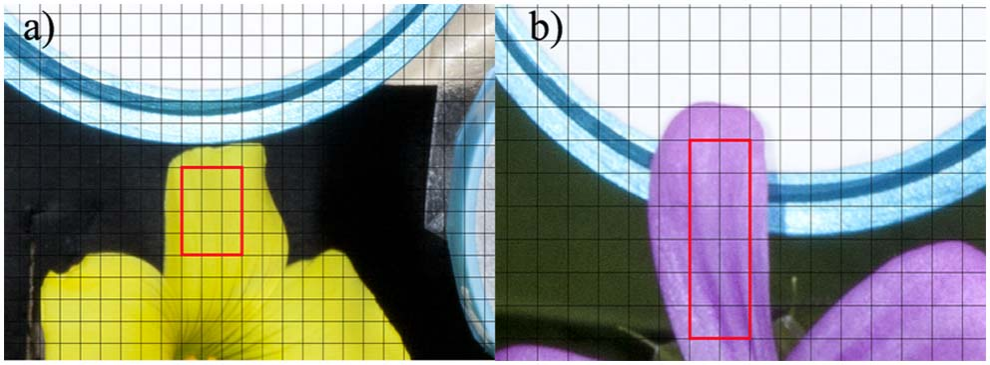
Examples of the two different grid schemes employed to measure local chromatic variability within a petal. a) ***Oxalis pes-caprae*** 4
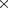
3 sampling scheme, grid size 40 pixels. b) ***Geranium sp.*** 6
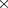
2 sampling scheme, grid size 30 pixels.

For the point sampling experiment, 15
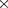
15 pixel areas were sampled from linearised images recorded using visible (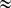
400 to 710 nm), and UV (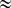
320 to 395 nm) radiation reaching the sensor of the Canon and Fuji cameras respectively. For the local variability experiment, three different square pixel areas were pseudo-randomly selected from either a 4
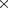
3 (sampling scheme A), or a 6
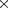
2 (sampling scheme B) square grid. The sampling scheme was selected so that a single petal of each one of the measured species was totally enclosed by the grid as depicted in Figure 5.

## Results

### Evaluation of the Linearisation Function

**Figure 6:**
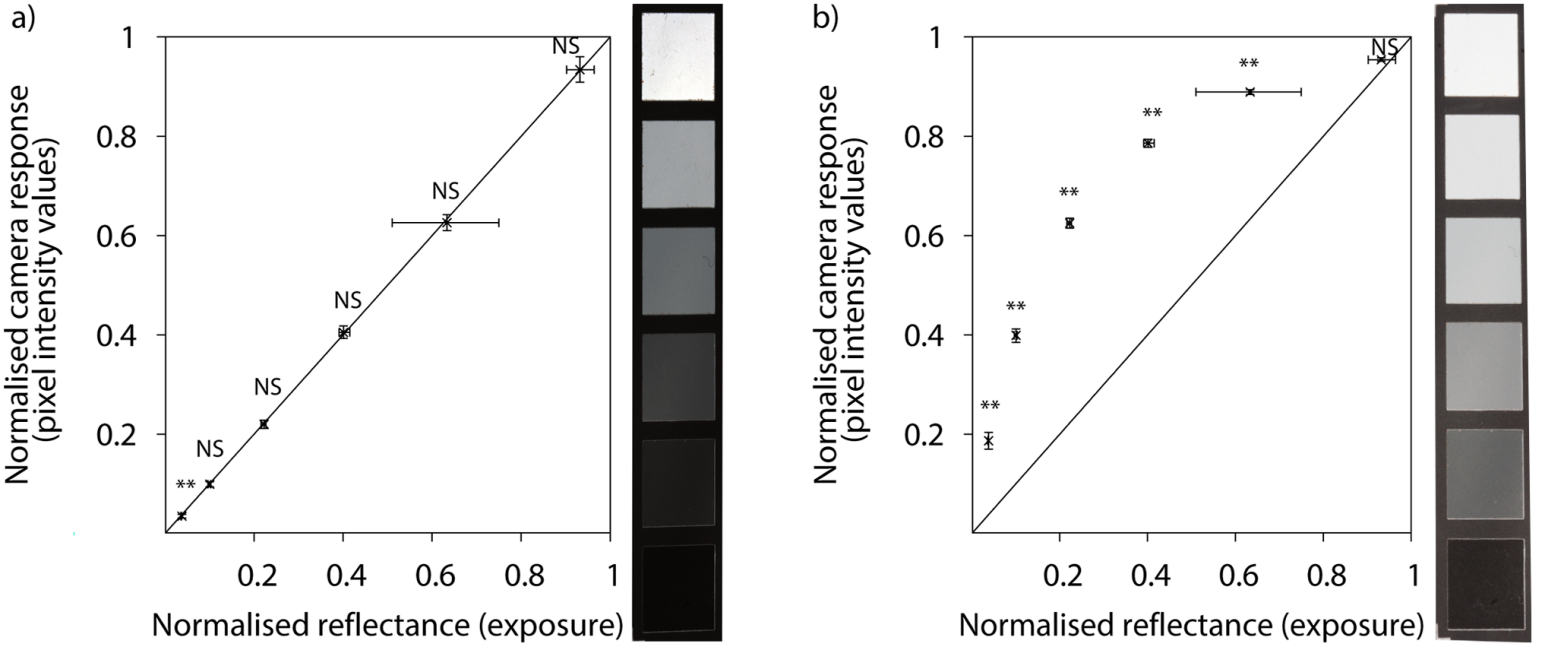
Linear (panel a) and non-linear (panel b) camera responses to a set of six achromatic samples from an X-Rite Colour Checker Passport corresponding to the green channel of a Canon 40D camera. The six achromatic samples uniformly reflect, from bottom to top, 3.10%, 9.11%, 19.5%, 37.2%, 60.9% and 94.8% of incident visible irradiation [47]. Camera responses correspond to an area of 900 pixels^2^ located at the centre of each grey swatch. Error bars along the *x*-axis represent pixel intensity variation within the sampling square, whilst error bars along the *y*-axis represent variation within recovered linear values arising from the uncertainty associated with the use of a biexponential linearisation equation [31]. Error bars represent standard deviation in both cases. 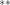
P-value significant at 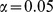; NS P-value not significant at 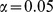.

Accuracy of the linearisation procedure was tested by comparing the recovered normalised linear camera responses obtained from the six achromatic samples present in the X-Rite Colour Checker passport against their reflectance values measured with a standard spectrophotometer (Figure 6).

Reflectance values obtained from the linear camera responses did not differ from those obtained by spectrophotometric readings for the achromatic samples reflecting from 9% up to about 95% of incident irradiation 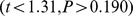. Reflectance values predicted from linear camera responses for the ‘black’ swatch, reflecting 3.10% of the incident radiation, did significantly differ from data published for this sample 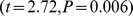 (Figure 3, panel a). For the non-linearised camera responses, all but those responses corresponding to the brightest achromatic sample 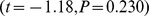 were significantly different from the responses predicted from spectrophotometric readings 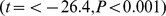 (Figure 3, panel b).

Images representing linear camera responses were always darker than their unprocessed, non-linear counterparts (Figure 6 image stripes and Figures 2–3). Reduced brightness in linearised image results from displacing the compressed camera responses at high irradiance levels (Figure 6, panel b) towards the middle region of the transfer curve (Figure 6, panel a).

### Quantitative Evaluation of Floral Chromaticity

Images representing linear camera responses in the visible and UV spectral regions were reconstructed from floral specimens of the species: *Oxalis pes-caprae, Goodenia ovata, Gazania rigens, Geranium sp., Malus domesticus, Freesia laxa, Eremophila macculata* and *Sonchus oleraceus* (Figures 2 and 3). On each image an achromatic, spectrally-flat standard was included as an internal control for exposure calibration and potential variations in colour that might have arisen as a result of the independent processing of each colour channel of the different images.

Total reflectance as measured by the camera was obtained from three 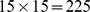 pixel^2^ sampling areas located at the tip, middle and base of a single petal of each one of the selected species, and from wider sampling areas (mean = 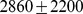 pixel^2^), representing wider areas than those covered by the spectrophotometer probe.

**Figure 7:**
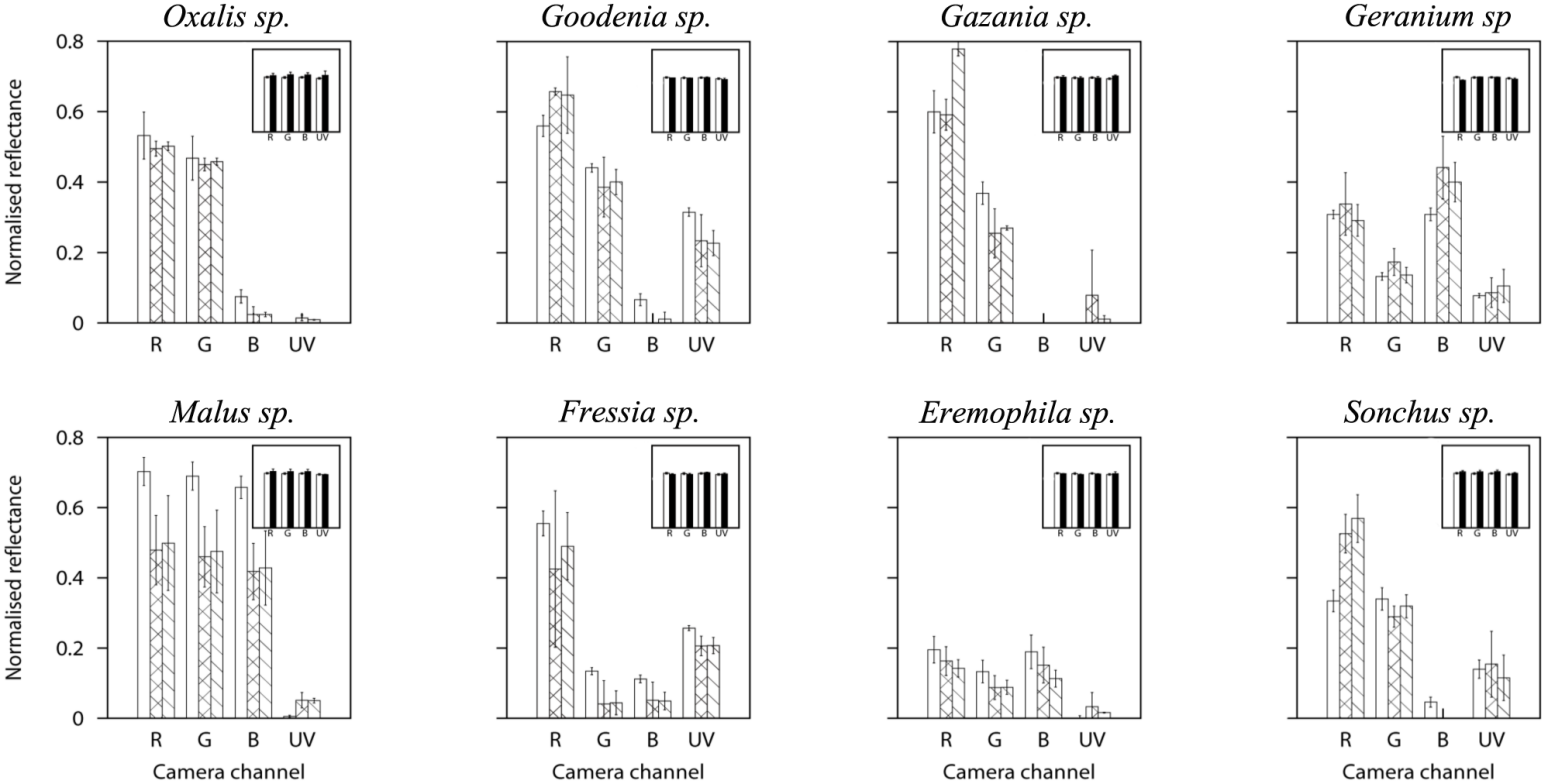
Total reflectance recorded from flowers belonging to the genera *Oxalis pes-caprae*, *Goodenia ovata*, *Gazania rigens*, *Geranium sp.*, *Malus domesticus*, *Fressia laxa*, *Eremophila macculata*, and *Sonchus olereaceus* by the different colour channels of a Canon 40D and a Fuji S3 UVIR. Expected camera responses, in reflectance values, calculated from spectrophotometric data and the spectral sensitivity curves of each colour channel (Figure 1) (white bars), camera responses for three 225 pixel^2^ areas located at the tip, middle and bottom of the petals (cross-hatched bars), and camera responses from sampling areas wider than those covered by a 400 *µ*m standard spectrophotometer probe (diagonal-hatched bars). Figure inserts represent measured and predicted camera responses for a spectrally flat, achromatic standard reflecting about 33% of incident irradiation, which was included as an internal control on each image. Error bars represent standard deviation in all cases.

In most of the cases the two methods differed in the magnitude of the mean total reflectance measured for the flower samples across the different spectral bands (Figure 7), but they were not statistically different for the achromatic calibration standard (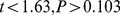). Moreover, the magnitude of the standard deviation from the intensity values obtained from linear camera responses was higher than that corresponding to the measured reflectance spectra (Figure 7). These results suggested that the differences observed in intensity values between the two methods are produced by the intrinsic local spatial variability of the samples rather than an inaccurate recovery of total reflectance from RGB responses.

To obtain a better understanding of the nature of the observed differences, we performed a new comparison between the total reflectance obtained from the two methods by sampling a wider area of *Goodenia ovata* incorporating the three lower petals of the flower and including the UV-reflective marks (Figure 4, panels a and b). For this measurement, the number of samples measured with the spectrophotometer was increased to 15 and the image sampling area was increased to cover an area of 2500 pixels^2^ matching that sampled with the spectrophotometer.

Total reflectance calculated from spectral data and recovered by the camera system is graphically summarised in Figure 4 panel c, along with the measurements obtained from the grey calibration standard. Consistent with data in Figure 3, total reflectance values obtained from spectrophotometry and from linear camera responses significantly differ from one another for the red, green and blue channels of the Canon 40D (Wilcoxon rank sum test 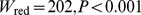, 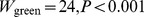, 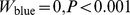); however, we did not find significant differences in the amount of total reflected UV measured by the two methods (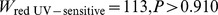).

**Figure 8:**
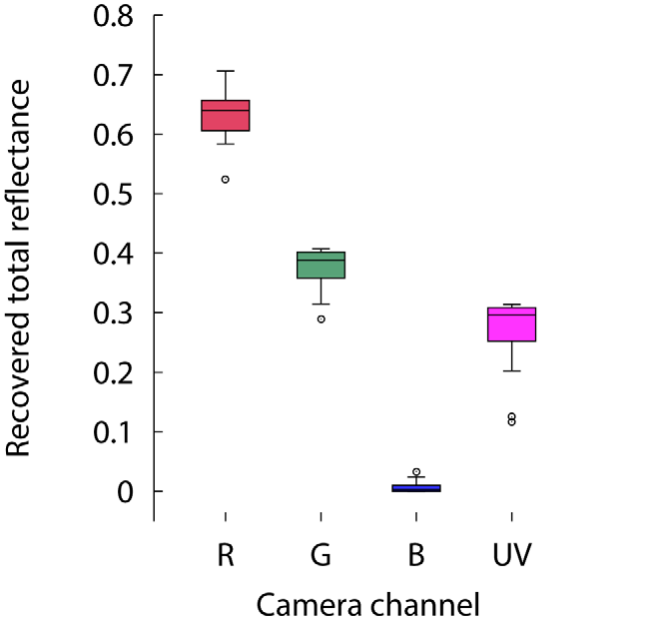
Graphical summary of the mean total reflectance recovered from fifteen 900 pixel^2^ square sampling areas covering the entire lower petals of a floral specimen of *Goodenia ovata* by the red, green and blue channels of a Canon 40D camera sensitive to visible radiation and the red UV-sensitive channel of a Fuji S3 UVIR camera.

Results from the last experiment suggest that chromatic variability is not equal across the different spectral bands but higher in some spectral regions. In the case of *Goodenia ovata*, the lowest variability was observed in the UV-sensitive channel and highest in the long wavelength (red) channel (Figure 4, panel I). Distributions of the total brightness values for each colour channel are summarised in Figure 8. Total reflectance values recovered by all but the red channel of the Canon 40D camera were found to be significantly non-normal at an 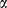 level of 0.05 (Shapiro-Wilk 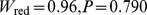, 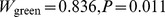, 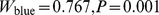, 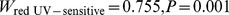).

## Discussion

### Flower Spatio-chromatic Variability

Naturally occurring samples typically present wide variations in their total reflectance values across two dimensions (Figure 4). This variation arises from intrinsic characteristics of each sample such as pattern texture, shape and volume (Figure 2–3). These characteristics are often overlooked, probably because of the difficulty of measuring them accurately using point samples [49], [50]. Yet measuring this variation is potentially of biological importance since it provides insight into the challenges faced by a pollinator’s visual system for detecting and correctly discriminating target flowers in visually complex environments. Our finding is consistent with a recent report that spectral measurements from different flowers of the same species often show wide variability in spectral signals to pollinators [19].

Misrepresentation of visual complexity when using spectrophotometry may arise as point samples can not always represent potential variation in shape, pigmentation and/or lighting effects for a given flower. For example, the ‘white’ flower of *Malus domesticus* has a complex three dimensional structure resulting in self-shading effects that the visual system of a pollinator would have to deal with (Figure 3). A spectrophotometer measuring colour at an ideal angle would potentially underestimate spectral signal variability (Figure 7). Thus the variability in the different systems reported here should be taken as a representation of the likely differences in signal processing attainable with a typical spectrophotometer set-up and a digital camera system with a limited number of spectral bands (*n* = 4) and staged in a consistent manner. This can be clearly seen by comparing the variability of colour signals within boxes of Figure 7, which illustrate the greater variation in signal in *Geranium sp.* than in *Oxalis pes-caprae*.

The appearance of flowers may also be influenced by various optical effects like iridescence [51], [52] produced by microscopic structures acting as photonic crystals or diffraction gratings [53]. Presence of iridescence in a petal or other plant material may increase the intensity of a given colour signal compared to a pigment-based signal [52], [53], which may lead to overexposed or clipped RGB values. The presence of these signals will undoubtedly introduce artefacts in the estimation of the signal’s radiance by means of linear camera responses [26]. This is one reason for employing specialised methods for the measurement of these particular signals [53], [54].

Variability on any given flower is not only limited to fluctuations in total brightness across the sample, but it is also manifested within the different spectral bands (Figure 8). This chromatic variability is represented by discrepancies in the shape of the distributions of the linear pixel intensity values within the UV, blue, green and red spectral bands, and are likely produced by the presence of pattern elements of varying hue and intensity within a petal. For instance, the highest chromatic variability in *Goodenia ovata* was observed in the red and green colour channels, corresponding to the (human-perceived) yellow pattern, whilst the lowest variability corresponded to the plain UV-reflective marks (Figure 4).

Other sources of spatial variability may also include variations in the recorded reflectance values produced by volume scattering, which is characteristic of leaves, fine plant structures and small dust particles [55]. Although the effect of volumetric scattering has been studied for radar imagery and other remote sensing techniques [55], [56], its potential effects on measurements using purely optical radiation remains untested. Future work could address this issue, in particular, when using infrared digital imaging.

Lack of uniformity in the brightness values through the sampled area and the selected spectral bands, suggests that visual signals produced by flowers are complex and should be regarded as multidimensional entities where each dimension potentially represents a different source of variability.

### Considerations when Using Consumer-level DSLR for Measuring Flower Colours

In spite of the advantages of digital photography to measure spectral and spatial variation in flower signals, recovered linear camera responses are limited to: *i)* the uncertainties associated with the implementation of a biexponential linearisation equation, *ii)* the colour gamut covered by the camera’s own colour space and *iii)* the spectral interval spanned by sensitivity curves of the colour channels available in the camera system.

The uncertainty associated with the recovered linear response is not uniform along the different values of the camera response [31], being particularly high at 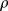 values corresponding to less than about 9% of incident radiation (Figure 6). Below this point camera responses are dominated by noise and are very likely described by a different relationship than camera responses at higher irradiance levels [26], [57].

In spite of being qualitatively close to the perceived aspect of a flower by a human observer, camera responses corresponding to highly saturated, human-perceived yellow and orange colours, such as those displayed by *Gazania rigens* and *Sonchus oleraceus*, do not correspond to those expected from their reflectance spectra; in other words, camera responses are not radiometrically faithful for these hues. This problem arises due to the smaller colour space reproduced by digital devices compared to animal colour spaces and that of a human as described by the CIE observer [48], [58].

It is likely that in order to reproduce a colour perceptually similar to that observed in highly saturated yellow samples, the inbound camera software increases the red channel response above the physical reflectance value for these samples whilst lowering the response of the green channel (Figures 2–3 and 7). As a consequence, linear camera responses corresponding to these hues are likely to be inaccurate. An efficient way to evaluate this potential problem is by measuring the RGB values in the non-linear image. Values corresponding to the red channel for the sample should never be above those reported for an achromatic calibration target reflecting more than about 90% incident irradiation. However with the current data it is not possible to identify the precise mechanism by which this correction is made, or if it involves a linear or non-linear transformation. A better understanding of the way the camera software deals with these colour samples remains an open field for research, and could be of value for other image processing tasks involving linear camera responses such as spectral reconstruction [59].

Finally, caution is suggested when interpolating linear camera responses to other trichromatic colour spaces, including CIE uniform colour spaces such as the CIE *Lab*, as the spectral tuning of the two systems may not completely overlap [60]. For example, a direct mapping between the Canon 40D camera employed here and any colour space based on human vision is very likely to be inaccurate as the spectral tuning of the respective systems differs (Figure 1). This limits the number of visual signals that can be accurately mapped to those whose spectral signature matches the spectral interval shared by the sensitivity functions of the two systems predicted by Equation 1, and has motivated the development of methodologies for spectral reconstruction from RGB camera responses [27], [60]. Future work should aim to obtain a better understanding of mappings between camera colour space and those of different animal observers through carefully designed camera characterisation experiments.

## Conclusion and Recommendations

Most consumer-level RGB cameras constitute an adequate means to recover total energy reflected within a spectral interval equal to that spanned by the spectral sensitivity of each channel, which, for certain specialised models, may include the UV region of the spectrum. However, the accuracy of recovered reflectance information is dependent on a proper characterisation process, including modelling of the unique transfer function of a specific camera, and the formulation of a linearisation equation.

Linear camera responses may be used to assess the intrinsic two-dimensional chromatic variability of naturally-occurring objects due to the simultaneous measurement of many points, each represented by a single pixel within an image. The measurement of spatio-chromatic variability allows this system to gauge the complexity of natural environments, thus giving researchers an insight into the challenges met by animal visual systems. Future applications could include accurate mapping of variability in natural backgrounds.
